# Physicochemical, Microbiological and Technological Properties of Red Deer (*Cervus elaphus*) Milk during Lactation

**DOI:** 10.3390/ani11030906

**Published:** 2021-03-22

**Authors:** María Isabel Berruga, Juan Ángel de la Vara, Carmen C. Licón, Ana Isabel Garzón, Andrés José García, Manuel Carmona, Louis Chonco, Ana Molina

**Affiliations:** 1 Food Quality Research Group, Institute for Regional Development (IDR), Universidad de Castilla-La Mancha, 02071 Albacete, Spain; juanangel.vara@uclm.es (J.Á.d.l.V.); manuel.carmona@uclm.es (M.C.); ana.molina@uclm.es (A.M.); 2 Department of Food Science and Nutrition, California State University, Fresno, 5300 N Campus Drive M/S FF17, Fresno, CA 93740, USA; cliconcano@mail.fresnostate.edu; 3 Departamento de Producción Animal, Universidad de Córdoba, 14071 Córdoba, Spain; pa1gasia@uco.es; 4 Animal Science Techniques Applied to Wildlife Management Research Group, Instituto de Investigación en Recursos Cinegéticos (IREC), Albacete Section of CSIC-UCLM-JCCM, Universidad de Castilla-La Mancha, 02071 Albacete, Spain; andresjose.garcia@uclm.es (A.J.G.); louis.chonco@uclm.es (L.C.); 5 Sección de Recursos Cinegéticos y Ganaderos, Institute for Regional Development (IDR), Universidad de Castilla-La Mancha, 02071 Albacete, Spain

**Keywords:** red deer, milk, lactation, somatic cells, microbiology, viscosity, color, particle size, coagulation

## Abstract

**Simple Summary:**

Milk from red deer is richer in fat and proteins than that of cow or other ruminants. The semi-captive breeding of this species has traditionally focused on meat, velvet or hunting purposes, but recent studies suggested that the high level of nutrients, the promising content of bioactive peptides and the better digestibility than that of milk from other species could open innovative alternatives for the dairy industry. As for other non-commercial mammalian species that are gaining technological interest for the elaboration of dairy products, it is necessary to understand the aptitude and performance of milk from red deer to be used for the production of cheese, fermented milks or other products. Our study aims to assess some chemical, physical, microbiological and technological properties of red deer milk during a lactation period of 18 weeks. The results show that milk from this species is similar to that of other ruminant species whose milk is commercialized. In addition, our results indicate the best period to industrialize the milk during lactation. To the best of our knowledge, this is the first study to explore the benefits of using red deer milk with a technological approach.

**Abstract:**

This study describes chemical, physical, microbiological and technological characteristics of red deer milk and the effect of lactation on these parameters in order to know their potential aptitude to elaborate dairy products. During 18 weeks, milk from five hinds was monitored for composition, bacteriology, somatic cell count (SCC), physical properties and rennet coagulation. Mean values (g/100 g) for fat, protein, lactose and dry matter were 10.4, 7.1, 4.3 and 24.2, respectively, and for urea, 265 mg/100 mL. Except for lactose, a significant increase in these components was observed (*p* < 0.01) as lactation progressed. The average values for bacteriology and SCC were 5.3 log cfu/mL and 4.7 log cells/mL, respectively. Regarding physical properties, conductivity (mean: 2.8 ms/cm), viscosity (3.1 Cp), coordinates L* (89.9) and a* (−3.1) and milk fat globule diameter (D_4,3_: 6.1 µm) increased along with lactation while density (1.038 g/mL) decreased (*p* < 0.01). The pH (6.7), acidity (22.9° Dornic), coordinate b* (8.4) and ethanol stability (66.6% *v*/*v*) were stable during the study period. The stage of lactation also has a significant impact on milk coagulation properties and mean curd yield was 3.29 g/10 mL. These results suggest that red deer milk could be a potential innovative source of milk for the dairy industry.

## 1. Introduction

Knowledge of the properties of milk from non-commercial mammalian species has been traditionally approached more from the viewpoint of lactation biology or physiology than from a technological perspective [1]. Apart from cow, buffalo, sheep, goat or camel milk, a marginal production of milk from other species, even some wild species, is described by Claeys et al. [2] as potential dairy animals. These authors emphasized the revival of milk from horse, donkey or some camelids in many countries because of their potential in cosmetics or therapeutics and revised the nutritional value of milk from several deer species. In fact, there is a growing interest in knowing about and using milk from these species as innovative alternatives for the dairy industry. In recent decades, studies on the chemical and microbiological quality or application of milks from Equidae or non-commercial ruminants to elaborate fermented milks have increased [3,4,5].

Red deer (*Cervus elaphus*) has usually been farmed for meat, velvet or hunting purposes, but in recent years, the potential for developing dairy farms is increasing, and some references about the commercialization of red deer dairy products and cosmetics have been reported [6,7,8,9]. Several countries such as New Zealand, Spain and even the USA have a high number of farms or deer parks either for meat or hunting (or both) in which the animals are raised in semi-captive conditions [10,11,12], making future breeding of deer with a dairy purpose more feasible. In fact, cosmetic products that include this milk are already marketed by the pharmaceutical South Korean Yuhan Corporation under the “Deerest” brand, and Gouda and Havarti-type cheeses are also marketed by the New Zealand company “Talbot Forest Cheese” reaching market prices of around NZD 200 per kg [13]. In the same country, the “Pāmu Farms” company has been producing and exporting powdered and liquid milk since 2015 for cosmetic products and restaurants [8,14]. Although there are no official figures about red deer milk production, if we consider the figures reported by New Zealand entrepreneurs of between 5000 and 6000 L of milk a year and with a flock of 70–80 hinds [14], a potential production of around 60–75 L/animal could be estimated without compromising the breeding of calves. As red deer is not yet a fully domesticated species, as indicated by Wang et al. [9], there are still many handling aspects that need to be solved to obtain commercial red deer milk—for example, milking mechanization, seasonality and milk production optimization, among others. However, the experience of New Zealand, a country with more than 2500 farms and a population of more than 400,000 mated hinds, suggests a promising opportunity for deer farmers to be exploited all over the world [9,11].

Milk from this species is richer in fat and proteins than that from cows or other non-bovine milk. With a mean composition of 11.5% fat, 7.6% protein, 5.9% lactose and 26.7% dry matter (DM) [15] and a mineral mean concentration for Ca, K, Mg, Na, P and Zn of 1717, 860, 109, 233, 901 and 9.06 mg/kg, respectively [16], its nutritional potential is well demonstrated. Red deer milk is not the only one that contains a high fat and protein content among ruminants; in fact, other species such as reindeer, domestic buffalo or springbok (African antelope) also contain high concentrations of these components. The milk of reindeer is produced commercially and even contains higher fat (10–19%) and crude protein (7–13%) contents [17]. Moreover, domestic buffalo milk has a fat content of 7–12% [18], while the milk of springbok also has fat and protein contents around 14.5% and 7.4%, respectively [19]. Furthermore, recent investigations have also confirmed the importance of red deer milk as a promising source of bioactive peptides from whey proteins [6,9,20]. Other studies focused on the buffering capacity and in vitro digestibility of red deer milk, which is higher than that of cow milk [20,21], and on its gelation properties in the environment of the human stomach [22]. In addition, the peptides and whey proteins of red deer milk have shown immunomodulatory bioactive and health-promoting properties [20].

Landete-Castillejos et al. [15] recorded a total milk yield of 224 L during lactation that extended for 34 weeks. These authors calculated that the milk production for a standard period of 15 weeks was 147 L and a daily milk production of 0.9 L, but in this lactation period, milk gross composition changes significantly [15]. Another study by this group in conditions including better pasture indicated a mean production over 14 weeks of 229 L [23]. Their suitability for milking is good, and in fact, there are farms where mechanical milking has been carried out for several years with or without the need for anesthesia [15,24]. Recently, de la Vara et al. [25] studied the chemical composition, somatic cell count and immunoglobulin G (IgG) and M (IgM) contents in red deer colostrum during its transition to milk, considering the mature milk after 2 weeks. Therefore, the potential use of milk from this ruminant species for dairy product elaboration must be considered promising. In fact, some trials of cheese and yogurt production have lately shown good yields [26,27], suggesting its good aptitude. In this sense, it is of significant interest to establish the main physicochemical properties of red deer milk during lactation because variations in milk properties could suppose problems or new opportunities for the manufacturing of dairy products. Recently, a comparison of ethanol stability, a freshness and heat stability indicator, with other dairy species showed that the milk of red deer is closer to sheep milk than cow or goat milk [24], but information about other characteristics is scarce in red deer milk, making it necessary to know its properties during the lactation period in detail. The present work investigated not only the chemical but also the physical, microbiological and technological properties of red deer milk during a lactation period of 18 weeks.

## 2. Materials and Methods

### 2.1. Ethical Statement and Animal Management

This study was conducted in accordance with the Spanish legislation for the use of animals in research [28] and with the approval of the Ethical Committee in Animal Experimentation from the University of Castilla-La Mancha (Permit Number: 1002.04). Handling and sampling procedures were designed to minimize stress and health risks. Hinds and calves of the species *Cervus elaphus* from the Experimental Farm of the University of Castilla-La Mancha (Spain) were examined daily by farm personnel and weekly by an experienced veterinarian. Red deer hinds of an Iberian population were reared in captivity conditions in a 5000-m^2^ open-door enclosure on an irrigated mixed pasture. Animals were fed in a system simulating the feeding system of deer farms using the same feeding program (a concentrate and a total mixed ration) described by Serrano et al. [29], which was common for all animals and met or exceeded the nutrient requirements for late pregnancy and lactation of hinds recommended by the National Research Council [30]. Ingredients and proximate composition [31] of the concentrate and ration are shown in Supplementary Table S1. Feed and water were offered ad libitum throughout the trial.

### 2.2. Milk Samples and Milk Production

In 2016, during the calving season from May to October, raw milk from 5 red deer hinds (average age 9.2 ± 6.6 years) was sampled. For milking, hinds were isolated from calves from 10:00 to 16:00 h in a handling facility without pre-isolation for ethical issues [15]. Milking frequency was reduced to the minimum considered essential to prevent stress and calves could perform a natural rate of suckling except the 6 h when hinds were separated for milking. Hinds were milked using a machine milking set up to a 50/50 massage/milking ratio at 44 kPa of vacuum at weeks 4, 6, 10, 14 and 18 of lactation. The daily milk production (DMP, mL/day) was calculated by multiplying the milk production collected per milking by four [15,32]. Milk samples of 250 mL were collected and kept at 4 °C until analysis in laboratory (not longer than 12 h) or freezing for posterior analysis (not longer than 1 month).

### 2.3. Milk Composition, Bacteriology and Somatic Cell Count

Protein, fat, lactose, casein (CN), dry matter (DM) content and urea concentration were measured in duplicate in raw milk at the Interprofessional Dairy Laboratory of Castilla-La Mancha (LILCAM, Castilla-La Mancha, Spain) by using an infrared spectrophotometer (MilkoScan 4000, Foss, Hillerød; Denmark) calibrated for cow, goat and sheep milk and subjected to quality controls and interlaboratory trials with the Spanish network of official Dairy Laboratories. Samples were diluted by 50% with distilled water to adjust their composition to the calibration range of the analyzer [33]. These parameters are expressed as g/100 g. Additional information of somatic cell count (SCC) and total bacteria count (TBC) was also obtained in duplicate from the LILCAM analysis using a Fossomatic 5000 and BactoScan FC, respectively. Both parameters, SCC and TBC, were normalized and converted to log10 SCC and log10 TBC, respectively.

### 2.4. Physical Analysis

The pH was measured using a Crison model GLP 22 pH meter (Crison, Barcelona, Spain) with a 5211 probe (Crison), and the titratable acidity (° Dornic) was determined in milk using NaOH 0.111 M and phenolphthalein as an indicator. The conductivity of different milk samples was measured at 25 ± 0.5 °C using a conductivity meter (Crison model Basic 30) with an EC 5293 probe (Crison). To determine the density of raw milk, a lactometer (Berman) ranging from 1.020 to 1.040 g/mL was used. Measurements were carried out at 15 ± 1 °C. Milk viscosity analysis was carried out using a digital rotational viscometer (Fungilab, model Visco Basic Plus L), with a Low Viscosity Adapter LCP/B. The measurements were performed at 20 ± 0.5 °C, with a spindle rotation ranging from 200 to 250 rpm. The viscosity value was set by the average of 2 measures. Milk color was measured using a Minolta CR-400 colorimeter (Minolta Camera Co., Osaka, Japan) with a CR-a33f cone and a calibrated white plate (Minolta 11333110) with Y = 93.1, x = 0.3160 and y = 0.3323. A D65 illuminant and an angle vision of 10° were used. CIE L*, a* and b* coordinates were obtained, where L* corresponds to brightness, a* to the red–green component and b* represents the yellow–blue component. The color measurement was made in transparent polystyrene 60-mL bottles (Deltalab, Rubí, Spain) that contained 50 mL of the sample by introducing the colorimeter 2 mm in the liquid and using a white background. Duplicate measurements for every sample at each sampling time were taken.

### 2.5. Ethanol Stability

Ethanol stability was measured by mixing equal volumes (1 mL) of the milk sample and the ethanol solution (water/ethanol ranging from 10 to 100% at 2% intervals, *v*/*v*) at room temperature. The maximum concentration of the ethanol solution which did not cause coagulation was defined as the ethanol stability of milk. Each sample was evaluated by three observers on the visual scale.

### 2.6. Milk Fat Globule Size

The milk fat globule diameter (MFG) was determined by Laser Light Scattering (LLS) using a Mastersizer 2000 analyzer (Malvern Instruments, Malvern, UK) and Hydro 2000SM (A) software version 2.0. To determine the fat globule size distribution, milk was mixed in a ratio of 1:1 with 35 mM EDTA, pH 7.0, to disrupt the casein micelles. The obscuration rate was kept between 8 and 16%. The refractive indexes for milk fat and water were 1.46 and 1.33, respectively. The absorption coefficient at both wavelengths was 0.0001. All analyses were performed in triplicate at room temperature. According to Ménard et al. [34], standard parameters were calculated using the software as follows: (a) the volume-weighted mean diameter D_4,3_ was used as the measure for the average MFG size and was defined as *∑*n_i_d_i_^4^/*∑*n_i_d_i_^3^, where n_i_ is the number of fat globules of diameter d_i_; (b) D_3,2_, is the volume surface average diameter and was defined as *∑*n_i_d_i_^3^/*∑*n_i_d_i_^2^; (c) the specific surface area (SSA) was defined as 6φ/D_3__,__2_, where φ is the volume fraction of milk fat; and (d) the span or size distribution width was defined as d_(0_._9)_−d_(0_._1)_/d_(0_._5)_, where d_(0_._9)_ is the diameter below which lies 90% of the globule volume—10% for d_(0_._1)_ and 50% for d_(0_._5)_.

### 2.7. Rennet Coagulation

Coagulation properties were measured according to Caballero-Villalobos et al. [35]. For that, a group of 20 hinds was used to obtain pools composed of a mix of milk from 6–7 hinds in their 4th, 6th, 8th and 10th week of lactation (50 mL/animal). Three pools were obtained for each lactation stage at different weeks. Samples from pools were preheated at 32 °C and renneting parameters were monitored using a Formagraph viscometer (Foss Electric, Hillerød, Denmark) based on the oscillatory motion of circular pendula immersed in the milk during coagulation. The testing time of the analysis was set to 60 min and the measured parameters were rennet clotting time (*r*, expressed in min), curd firming time (k20, in min) and curd firmness after 30 and 60 min (A30 and A60, respectively, expressed in mm). Milk samples that did not coagulate within 60 min after rennet addition (*n* = 1) were considered missing. Curd firmness was also considered missing when the result was 0 (A30: *n* = 1; A60: *n* = 1). To measure curd yield (g/10 mL), curds were individually placed in centrifuge tubes, cut with a spatula and centrifuged (30 min, 2800× *g*, 37 °C) to separate the whey.

### 2.8. Statistical Analysis

A one-way analysis of variance was performed using SPSS 24.0 (IBM SPSS Inc., Chicago, IL, USA), where the lactation time was the categorical factor used to explain variations of the parameters studied. Tukey’s test at a significance level of *p* ≤ 0.05 was used to determine differences in each parameter with the period lactation time.

## 3. Results and Discussion

### 3.1. Milk Production and Chemical Red Deer Milk Properties

Table 1 presents the mean values and lactation period effect for daily milk production (DMP) and the gross composition of the raw milk collected from five individual red deer hinds over the period from May to October 2016. DMP decreased significantly (*p* < 0.000) during lactation, with mean values similar to those reported by other authors at the same lactations times [29,33]. Mean red deer milk values were 10.4, 7.1, 5.1, 4.3 and 24.2% for fat, protein, CN, lactose and DM, respectively. As for other mammals, fat was the milk component that had higher variations in the 18 weeks of study, increasing significantly by more than 3.5% from the first to the 18th week. Total protein and CN concentrations also increased significantly as lactation progressed, while lactose content remained stable. In the case of urea, with a mean value of 265 mg/100 mL, a significant drop after the tenth week was observed. Considering that, over the study, hinds were fed equally and that protein concentration increased as a consequence of a milk concentration effect, this decrease in urea at the end of the lactation could be explained by a lower protein intake by hinds in these last weeks, coinciding with the hottest period during lactation. Studies on heat stress in hinds and calves at our farm have shown that increased heat stress leads to a lower growth in calves and, in the case of higher values of thermal stress indices, also in lactating females [36]. The literature on cattle shows feed intake reduction and lower protein in milk during high thermal stress in cows [37], but while the latter study showed no effect of heat stress on urea, Costa et al. [38] showed in buffalo that urea had one of the strongest correlations with heat stress indices, although a positive one and not the postulated negative one of our results. Unfortunately, our study was not designed to discern between the mixed effects of greater heat stress in the last weeks of lactation and the effect of reduction in milk production and increase in some nutrients such as protein or fat (maybe paired with a reduction in urea).

The values for milk composition were closer to those described by others [15,39,40] that also previously observed significant increases in fat, total protein, CN and DM over the lactation period. For all of these compounds, the increment was more pronounced in the last eight weeks. The average gross composition of red deer milk was higher in comparison with milk from cow, goat, equids or camelids (Table 2) and slightly higher than sheep or buffalo milk but similar to other cervids, such as reindeer or fallow deer [1,2,5,17,18,41]. The high content of fat and proteins suggests that red deer milk could be a suitable alternative for cheese production because of its great nutritional value and promising cheese yield.

### 3.2. Total Bacteria and Somatic Cell Count of Red Deer Milk

The total bacterial count (TBC) of red deer milk showed a mean value of 5.26 log cell/mL (Table 3). Microbial levels were higher in the first months of lactation and decreased at the end, probably due to the coincidence with the slight decrease in temperatures at the end of the summer period. These levels were slightly higher than the official levels fixed for cow milk [65,66] but admissible if comparing with limits established for milk from small ruminant species in the EU [65,66].

Regarding the somatic cell count, the data revealed that animals had good sanitary conditions during the study period (Table 3). Studies about SCC in red deer milk are scarce and limited to a few individuals [24,33]. During the interval being studied, an increase in SCC at the end was not observed, unlike the slight increase noticed for a similar period by Pérez Serrano [33]. Nevertheless, further studies with a higher number of individuals or bulk milk are necessary to have better knowledge of the physiological values for this species. In addition to the sanitary conditions of hinds, SCC is widely used for evaluating milk quality and could provide useful information of the impact on technological properties of milk. Several authors remarked that high SCC in sheep and goat milk has a great impact on the renneting and acidification properties of the milk, on cheese yield and on the composition and sensorial characteristics of cheese and yogurt [67,68,69].

If comparing with cow milk, where the official limit established by the European Union (EU) [65] and the US [66] regulations for cows is higher (5.6 and 5.87 log cells/mL, respectively) than counts observed for red deer milk, we could conclude that red deer milk fits within the cow milk standards setting in the EU and the US. Moreover, comparison with the limits established for sheep and goat milk also revealed that red deer milk meets the US standards (legal SCC limit of 1000 × 10^3^ cells/mL). Official limits for these small ruminant species in the EU have still not been regulated, but the threshold levels recommended by several European authors [70,71] suggest that this species could be considered within the standards of small ruminants.

### 3.3. Physical Red Deer Milk Properties

Mean values for the pH and acidity of the raw red deer milk were 6.73 and 22.96, respectively (Table 4). The range of pH (6.79 to 6.65) was narrower than the one described by Krzywinski et al. [40] during lactation of two hinds (6.25 to 7.15). These values of pH were similar to those found in commercial dairy species [41,46,56] or other mammalian species [64,72] (Table 2). The values for acidity were close to those of sheep milk and higher than those of goat, cow or buffalo milk [41,46,56] (Table 2). As for other species [46,72], the pH and acidity values did not change significantly over the 18 weeks of control, but a slight tendency toward acidification was appreciated when lactation progressed. This evolution of pH was different to that described by Krzywinski et al. [40], where pH increased with lactation. Bacterial content was normally attributed to milk acidification, but the evolution of pH and titratable acidity do not appear to be related to the bacterial content during lactation, with lower counts at the end of lactation. In addition, as all samples were analyzed in less than twelve hours, we can assume that acidity parameters mainly expressed the natural acidity and undeveloped acidity.

By contrast, milk electrical conductivity (EC) significantly increased, starting from the tenth week during lactation, from 2.31 to 3.56 mS/cm (Table 4). These values were higher than those described for buffalo [18], similar to those for sheep [24,41,59] and lower than those for goat and cow milk [24,41,57] (Table 2). A slight increase in the EC during lactation has also been observed in goat and cow lactations [57,58]. The information of electric conductivity of milk has been used to predict and detect subclinical and early clinical mastitis in other ruminant species [57]. However, as the SCCs observed at the end of lactation in the tested animals tended to decrease slightly as lactation progressed (Table 3), mineral composition could have more of an influence on the increase in EC than subclinical mastitis. In healthy goats, Díaz et al. [57] attributed this increase at this phase of lactation to changes in the blood–milk barrier due to a decrease in the tightness of the mammary epithelium that allows a greater permeability of Na^+^ and Cl^−^ from blood to milk. This is in agreement with the significant increment in Na^+^ as shown by Vergara et al. [16] with the progress of red deer’s lactation.

The average density of red deer milk was 1.038 g/mL and significantly decreased during lactation (Table 4). Comparable density values were reported by Krzywinski et al. [40]. The density of red deer milk is closer to sheep or buffalo milk, but is higher than goat and cow milk [41,46,56]. As for goat or sheep milk [56], the evolution with lactation tends to decrease, which could be explained by the significant increase in fat content at the end of lactation (Table 1).

The analysis of viscosity showed mean values of 3.12 cP (Table 4), comparable to sheep milk (2.5–3.9 cP) and higher than in dromedary camel (1.7–2.3 cP), cow (1.7–2.5 cP) or goat (2.1–2.2 cP) milk [49,64,73] (Table 2). The red deer milk viscosity increased significantly starting from the tenth month, in a correlating trend with the increment in fat and protein contents (Table 1). The change in milk viscosity depended on the contents of the milk: fat, protein and, particularly, the concentration and state of casein micelles [47], as viscosity is positively correlated with these components.

Regarding color, the mean value for the L* coordinate was 89.94 (Table 4), similar to values reported by others for deer milk [24,42] and slightly higher than the brightness values found in sheep and goat milk [24,60] (Table 2). Brightness increased (*p* < 0.002) with lactation, concurring with the increase in fat and protein contents (Table 1). Several authors have perceived a brightness increment with higher fat content in cow and sheep milk [48,60]. A direct influence of proteins, especially caseins, on the increasing brightness has also been reported [43]. Redness (a* coordinate) values ranged from −3.59 to −2.57 and increased during lactation (Table 4). These trends are closer to those found in sheep milk with different fat contents [60]. The mean value for yellowness (b* = 8.38) agrees with those found in other studies for deer milk [24,42]. The comparative observation of red deer milk yellowness with other species showed that this milk is yellower than sheep, goat, cow, camel or mare milk [24,42,60] (Table 2). These differences could be caused by the chemical differences between the milk for these species. In milk from other species such as sheep, a negative correlation for yellowness and fat content was observed [60], but although a marked increase in fat was observed at the end of lactation, yellowness tended to be stable (*p* > 0.05) during lactation in red deer milk (Table 4). These differences might be due to a different assimilation of carotenes and other natural pigments [42].

The ethanol stability (ES) did not change significantly during lactation—a mean value of 66.64% was obtained (Table 4). This value was close to that observed in buffalo and sheep milk (63%), but quite different from cow (83–93%) or goat (44–50%) milk [18,24,50,51]. The importance of ethanol stability (ES) for the industrialization of red deer milk lies in the fact that it is an indicator of freshness and provides information about heat stability in dairy processes. This is the reason why several countries, such as Spain [52], have established official limits for this parameter in raw milk. A more detailed understanding of ES for red deer milk has been published by de la Vara et al. [24].

### 3.4. Milk Fat Globule Size

The parameters calculated from the size distributions of MFG for red deer milk during lactation are shown in Table 5. The average volume-weighted mean MFG diameter D_4,3_ for this species was 6.12 µm—higher than those found for cow (2.5–5.7 µm), goat (2.76 µm), sheep (4.97 µm), buffalo (5 µm) or yak (4.19 µm) milk [34,53,74,75,76] (Table 2).

The larger size of red deer milk fat globules could be related with the higher percentage of fat found compared to other species [34]. Menard et al. [34] explained that the larger size could be due to a limitation of the fat globules’ membranes to envelop the synthesized fat during the secretion of the MFG from the epithelial cells of the mammary gland. This is also in agreement with a significant (*p* < 0.001) increase observed in the MFG diameter D_4,3_ during lactation as fat content increases (Table 1). By contrast, the rest of the size parameters were stable during lactation (*p* > 0.05). Red deer milk showed a mean D_3,2_ of 3.99 µm, a mean span of 1.25 and a mean SSA of 1.73 m^2^/g. Values for SSA for red deer milk were similar to those observed by others for cow, goat or buffalo milk that ranged from 1.71 to 2.17 m^2^/g [34,53] (Table 2).

### 3.5. Red Deer Milk Coagulation

Coagulation parameters for red deer milk are shown in Table 6. A clear difficulty for coagulation has been realized for milk pools corresponding to the fourth week of lactation; one sample did not coagulate after 60 min, rennet coagulation time (r) almost doubled and the time to curd firmness (k20) was six times longer compared with the next lactation time of sampling (*p* < 0.001). Consequently, after 30 and 60 min, values for A30 and A60 were also significantly lower (*p* < 0.001). These coagulation difficulties were also reflected in a lower curd yield at this lactation time (*p* < 0.05), which showed an average yield during lactation of 3.29 g/10 mL. The higher pH and SCC content in the first week of lactation (Table 3 and Table 4) could have some influence on this pattern [35,61,77]. After 6 weeks of lactation, the rennet coagulation time was reduced to around 25 min and was 16–17 min at the end of the analyzed period without significant changes. The mean value for *r* (25.23 min) was high compared with sheep (6.5–28.1 min), goat (12.9–13.2 min), buffalo (11.6 min) and cow milk (10–19.2 min) [35,44,45,54,55,61,62,63] (Table 2).

In the study period, the average curd firming time (k20) was 7.83 min, a much longer time than that observed in other species with values ranging between 1.57 min for sheep’s milk and 5.2 for cow’s milk [35,44,54,61,62]. However, if only milk from the sixth week onwards was considered, the values for k20 ranged from 2.27 to 3.56 min, times closer to those found in other species (Table 2). Thirty minutes after rennet addition, the mean A30 value was 20.73 mm, a value lower than that found in goat (36–44 mm), sheep (15–59 mm), cow (30–36 mm) or buffalo (40 mm) milk [35,44,45,54,55,61,62,63] before the first six weeks of lactation (Table 2). Nevertheless, after six weeks, the A30 values can be compared to those found in sheep, goat or cow milk. Results for A60 suggested that the red deer milk has different coagulation properties than other species because the distance between the oscillation width continues to increase, unlike what happens with other species where, at this point, the maximum values for A60 were reached and a progressive decrease is observed, indicative of syneresis [45]. To the best of our knowledge, the only coagulation pattern that is similar to the one previously described for red deer milk is found in Manchega ewes’ milk [35]. Nevertheless, as for other coagulation parameters, the lactation stage has a high influence as lactation evolves. Further research is needed to investigate factors that have effects on coagulation and cheese yield.

## 4. Conclusions

The red deer fresh milk obtained from captive hinds showed to be a good nutritional source for the manufacturing of dairy products. The results here indicated that red deer milk had good microbiological quality and the physical properties made this milk comparable with sheep milk, with similar acidity, conductivity, density, viscosity and ethanol stability, which suggested a good aptitude for being used in the processing of dairy products similar to those made with sheep milk. To the best of our knowledge, this is also the first time that red deer milk was studied using a technological approach, which has made it possible to know the noticeable effect of lactation stage on its rennet coagulation properties. Lactation stage had a significant effect on the gross composition, electrical conductivity, density, viscosity, color and size of milk fat globules but had no effect on acidity or ethanol stability, and these aspects should be considered when selecting the best lactation period to industrialize this milk. This work is a promising basis for the study and exploitation of red deer milk; however, more studies are needed for a better understanding of the effect of lactation on milk properties and protein coagulation by acid to elucidate its aptitude for fermented milk production. This will be the next step of our research.

## Figures and Tables

**Table 1:** Milk production and composition of red deer milk collected over the 18-week lactation period.

Parameter	Lactation Stage, Weeks ^1^					Mean	SEM	*p*-Value
	4	6	10	14	18			
DMP ^2^ (mL/day)	2362 ^a^	2545 ^a^	1795 ^ab^	1786 ^ab^	1313 ^b^	1960	106	0.000
Fat, g/100 g	9.00 ^a^	8.79 ^a^	10.26 ^ab^	11.24 ^bc^	12.70 ^c^	10.40	0.40	0.002
Protein, g/100 g	6.77 ^a^	6.76 ^a^	7.00 ^ab^	7.32 ^bc^	7.78 ^c^	7.13	0.11	0.002
CN ^3^, g/100 g	4.57 ^a^	4.67 ^a^	4.98 ^ab^	5.30 ^b^	5.97 ^c^	5.12	0.12	0.000
CN/Protein, %	67.4 ^a^	69.1 ^a^	71.1 ^b^	71.8 ^b^	72.65 ^b^	70.4	0.46	0.000
Lactose, g/100 g	4.17 ^a^	4.16 ^a^	4.34 ^ab^	4.28 ^ab^	4.72 ^b^	4.33	0.07	0.076
Urea, mg/100 mL	309 ^a^	320 ^a^	327 ^a^	163 ^b^	203 ^b^	265	14	0.000
Dry matter, g/100 g	23.15 ^ab^	22.71 ^a^	23.59 ^ab^	24.65 ^b^	26.76 ^c^	24.17	0.37	0.000

^1^ Means within a row with different superscript letters are significantly different (*p* < 0.05). ^2^ DMP: daily milk production; ^3^ CN: casein.

**Table 2:** Comparison of physicochemical and technological characteristics between different ruminant and non-ruminant species ^1^.

Parameter	Ruminants						Non-Ruminants	
	Red Deer ^2^	Cow	Goat	Sheep	Buffalo	Reindeer	Mare	Camel
Total solids, g/100 g	21.8–26.8	11.8–13.0	11.0–16.3	11.8–20.0	7.9–18.4	20.1–27.1	9.3–12.1	10.6–15.0
Fat, g/100 g	8.8–12.7	3.3–5.4	3.0–7.2	5.0–9.0	5.3–9.0	10.2–21.5	0.5–4.2	10.2–21.5
Total protein, g/100 g	6.7–7.8	3.0–3.9	3.0–5.2	4.5–7.0	2.7–4.7	7.5–13.0	1.4–3.2	7.5–13.0
Casein, g/100 g	4.6–5.9	2.6	2.4	4.2	3.5–4.6			8.3
Lactose, g/100 g	4.2–4.7	4.4–5.6	3.2–5.0	4.1–5.9	3.2–5.4	1.2–4-7	5.6–7.2	2.8–3.7
Urea, mg/100 g	163–320	393	- ^3^	-	-	-	-	-
pH	6.2–7.1	6.6–6.7	6.5–6.8	6.5–6.8	6.5–6.8	-	-	-
Acidity, °D	22–24	14–19	14–23	22–26	13–21	-	-	-
EC, mS/cm	2.1–3.9	4.7	4.2–5.9	3.9	0.7	-	-	-
Density, g/mL	1.037–1.040	1.023–1.031	1.028–1.035	1.035–1.038	1.030–1.035	-	-	-
Viscosity, cP	2.6–3.8	1.7–2.5	2.1–2.2	2.5–3.9	2.0–2.2	-	-	1.7–2.3
Coordinate L*	88.2–90.8	81.0–84.8	86.0	79.9–87.9	-	-	73.5	67.8
Coordinate a*	−3.6–2.6	−3.3–1.5	−2.1	−3.5–2.4	-	-	−2.2	−2.0
Coordinate b*	6.6–9.1	5.2–7.5	5.5	6.6–7.5	-	-	−2.3	−0.2
ES ^4^, % *v*/*v*	61–72	83–93	44–50	63	60–72	-	-	-
D_4,3_, µm	5.6–6.2	2.5–5.7	2.8	4.9	5.0	-	-	-
SSA ^4^, m^2^/g fat	1.6–1.8	1.9	2.2	-	1.8	-	-	-
*r*^4^, min	1.7–41.1	13.0–19.2	12.9–13.2	6.5–28.1	11.6	-	-	-
k20 ^4^, min	2.3–24.1	5.2	4.5	1.6–4.1	-	-	-	-
A30 ^4^, mm	1.0–34.6	30.1–36.0	36.0–44.0	15.1–59.3	40.2	-	-	-
A60 ^4^, mm	23.9–34.7	-	27.8	36.4–40.1	-	-	-	-
Curd yield, g/10 mL	3.2–3.4	-	-	-	-	-	-	-
References	[24,40,42]	[1,2,5,24,34,41,42,43,44,45,46,47,48]	[1,2,5,24,41,42,46,49,50,51,52,53,54,55,56,57,58]	[1,2,5,24,35,41,42,46,47,49,52,56,59,60,61,62]	[1,2,5,18,34,46,63]	[1,2,5,17,42]	[1,2,5,42]	[1,2,5,42,64]

^1^ Minimal and maximum content declared by different authors. ^2^ Including data obtained in this study. ^3^ no information for this parameter. ^4^ Abbreviations: ES—ethanol stability; SSA—specific surface area; r—rennet clotting time; k20—curd firming time; A30 and A60—curd firmness after 30 and 60 min, respectively.

**Table 3:** Total bacteria (TBC) and somatic cell (SCC) counts of red deer milk collected over the 18-week lactation period.

Parameter	Lactation Stage, Weeks ^1^					Mean	SEM	*p*-Value
	4	6	10	14	18			
TBC ^2^, log cfu/mL	5.37 ^b^	6.03 ^a^	4.91 ^c^	5.14 ^bc^	4.86 ^c^	5.26	0.10	0.000
SCC ^2^, log cell/mL	5.13	4.81	4.67	4.56	4.65	4.76	0.09	0.404

^1^ Means within a row with different superscript letters are significantly different (*p* < 0.05). ^2^ Abbreviations: TBC, total bacteria count; SCC, somatic cell count.

**Table 4:** Physical properties of red deer milk collected over the 18-week lactation period.

Parameter	Lactation Stage, Weeks ^1^					Mean	SEM	*p*-Value
	4	6	10	14	18			
pH	6.79	6.74	6.77	6.70	6.65	6.73	0.02	0.239
Acidity, °D	21.96	21.88	23.12	23.40	24.44	22.96	0.51	0.496
Electrical conductivity (EC), mS/cm	2.31 ^a^	2.29 ^a^	2.11 ^a^	3.90 ^b^	3.56 ^c^	2.83	0.15	0.000
Density, g/mL	1.040 ^a^	1.040 ^a^	1.037 ^ab^	1.037 ^ab^	1.034 ^b^	1.038	0.01	0.006
Viscosity, cP	2.95 ^ab^	2.83 ^a^	2.68 ^a^	3.36 ^bc^	3.76 ^c^	3.12	0.10	0.001
Coordinate L*	88.23 a	89.81 ^bc^	89.58 ^b^	90.79 ^c^	90.61 ^bc^	89.94	0.23	0.002
Coordinate a*	−3.59 ^a^	−3.32 ^a^	−3.58 ^a^	−2.87 ^ab^	−2.57 ^b^	−3.15	0.12	0.015
Coordinate b*	6.62	9.03	8.72	8.42	8.41	8.38	0.34	0.360
Ethanol stability (ES), % *v*/*v*	65.60	72.40	68.80	65.60	60.80	66.64	1.79	0.345

^1^ Means within a row with different superscript letters are significantly different (*p* < 0.05).

**Table 5:** Calculated parameters from the size distribution of fat globules in red deer milk during lactation.

Parameter	Lactation Stage, Weeks ^1^			Mean	SEM	*p*-Value
	6	10	18			
d_(0_._1)_ (µm)	2.23	1.62	1.81	1.80	0.12	0.167
d_(0_._5)_ (µm)	3.84	3.59	3.83	3.68	0.21	0.845
d_(0_._9)_ (µm)	5.88	6.10	6.34	6.22	0.29	0.884
D_3,2_ (µm)	4.19	3.84	4.24	3.99	0.12	0.320
D_4,3_ (µm)	5.62 ^a^	6.14 ^b^	6.26 ^b^	6.12	0.05	0.000
Span	1.10	1.31	1.18	1.25	0.07	0.665
SSA (m^2^/g fat)	1.61	1.78	1.71	1.73	0.05	0.285

^1^ Means within a row with different superscript letters are significantly different (*p* < 0.05).

**Table 6:** Coagulation parameters of red deer pool milk at different lactation periods.

Parameter	Lactation Stage, Weeks ^1^				Mean	SEM	*p*-Value
	4	6	8	10			
pH	6.77 ^a^	6.64 ^b^	6.39 ^c^	6.45 ^bc^	6.61	0.04	0.000
*r*, min	41.11 ^a^	25.64 ^b^	17.70 ^b^	16.67 ^b^	25.23	1.91	0.000
k20, min	24.07 ^a^	3.56 ^b^	3.12 ^b^	2.27 ^b^	7.83	2.46	0.002
A30, mm	1.00 ^a^	17.20 ^b^	29.69 ^c^	34.58 ^c^	20.73	2.56	0.000
A60, mm	23.95 ^a^	34.64 ^b^	32.86 ^b^	34.74 ^b^	31.93	1.08	0.000
Curd yield (g/10 mL)	3.18 ^a^	3.23 ^ab^	3.27 ^ab^	3.43 ^b^	3.29	0.03	0.011

^1^ Means within a row with different superscript letters are significantly different (*p* < 0.05).

## Data Availability

The data presented in this study are available on request from the corresponding author.

## Supplementary Materials

The following are available online at https://www.mdpi.com/2076-2615/11/3/906/s1, Table S1: Ingredients and proximate composition of red deer ration.
