# Supplementary material for: Physicochemical, Microbiological and Technological Properties of Red Deer (Cervus elaphus) Milk during Lactation
Source: Animals (Basel). 2021 Mar 22;11(3):906. doi: 10.3390/ani11030906 (PMC8004988; doi:10.3390/ani11030906)
Supplement: Supplementary file 1 [file animals-11-00906-s001.pdf]

Supplementary Table 1. Ingredients and proximate composition of red deer ration (%, on DM basis[1]).

Item	Concentrate	Total mixed ration
Ingredient composition	19.2	-
Corn	22.6	-
Wheat	11.3	-
Barley sprouts	13.6	-
Barley, 10.5% CP	7.7	-
Wheat bran, 20% starch	0.9	-
Sunflower mea., 28% CP	6.3	-
Palm kernel expeller	11.3	-
Soybean hulls	11.3	-
Alfalfa meal, 15% CP	4.5	-
Molasses, sugarcane	0.9	-
Grape seed	0.3	-
Palm oil	1.9	-
Calcium carbonate	0.7	-
Salt	0.5	-
Vitamin and mineral premix[2]	-	-
Oat	-	27.6
Alfalfa meal, dehydrated	-	48.4
Cereal Straw, from barley	-	20.8
Citrus Pulp, form orange	-	13.5
Proximate composition		
Moisture	11.6	30.1
CP	15.6	15.6
Ether extract	3.6	1.2
Crude fiber	15.3	34.9
ADF	20.0	38.6
NDF	35.6	57.2
Ash	7.9	10.1
Ca	1.1	1.6
P	0.5	0.2
Mn, ppm	117.8	30.5

[1] Unless otherwise indicated. [2] Supplied per kg of concentrate: vitamin A (trans-retinyl acetate), 10,000 IU; vitamin D3 (cholecalciferol), 2,000 IU; vitamin E (all-rac-tocopherol-acetate), 15 mg; Mn ($MnSO_4 \cdot H_2O$), 75 mg; Fe ($FeCO_3$), 50 mg; Zn ($ZnSO_4 \cdot H_2O$), 115 mg; I (KI), 2 mg; Cu ($CuSO_4 \cdot 5H_2O$), 7.5 mg; Se (Na_2SeO_3), 0.22 mg; Co ($2CoCO_3 \cdot 3Co(OH)_2 \cdot H_2O$), 0.83 mg; ethoxiquin, 0.025 mg; BHT (butilhidrotoluen), 0.18 mg; BHA (butylhydroxyanisole), 0.016 mg; sepiolite, 950 mg.
